# Supplementary material for: Emergence of Switch-Like Behavior in a Large Family of Simple Biochemical Networks
Source: PLoS Comput Biol. 2011 May 12;7(5):e1002039. doi: 10.1371/journal.pcbi.1002039 (PMC3093349; doi:10.1371/journal.pcbi.1002039)
Supplement: Text S1 — ODEs and parameter values for Fig. 6, and the method used in translating bistable network models into the experimental data mining format. (PDF) [file pcbi.1002039.s006.pdf]

Emergence of switch-like behavior in a large family of simple biochemical networks

Dan Siegal-Gaskins[1,2], Maria Katherine Mejia-Guerra[2], Gregory D. Smith[3], and Erich Grotewold[2]

1. Mathematical Biosciences Institute, The Ohio State University, Columbus, OH 43210, USA
2. Department of Molecular Genetics and Plant Biotechnology Center, The Ohio State University, Columbus, OH 43210, USA
3. Department of Applied Science, The College of William and Mary, Williamsburg, VA 23187, USA

Supplementary Text

ODEs and parameter values for Fig. 6

The dimer-free network shown in Fig. 6 can be described with the following set of ODEs:

$$P_1' = k_1*(X_1^{tot} - X_1P_2) - k_7*(X_2^{tot} - X_2P_1 - X_2P_2)*P_1 + k_8*X_2P_1 - k_{11}*P_1$$

$$P_2' = k_2*(X_2^{tot} - X_2P_1 - X_2P_2) - k_5*(X_1^{tot} - X_1P_2)*P_2 + k_6*X_1P_2 - k_9*(X_2^{tot} - X_2P_1 - X_2P_2)*P_2 + k_{10}*X_2P_2 - k_{12}*P_2 + k_{16}*X_2P_2$$

$$X_1P_2' = k_5*(X_1^{tot} - X_1P_2)*P_2 - k_6*X_1P_2$$

$$X_2P_1' = k_7*(X_2^{tot} - X_2P_1 - X_2P_2)*P_1 - k_8*X_2P_1$$

$$X_2P_2' = k_9*(X_2^{tot} - X_2P_1 - X_2P_2)*P_2 - k_{10}*X_2P_2$$

where the P_i are the concentrations of the free protein monomers, the X_iP_j are the concentrations of the protein-DNA complexes, the X_i^{tot} are the total DNA (free + bound) concentrations, and the k_i are model parameters. The bifurcation plot was generated with the following parameter values: $X_1^{tot} = X_2^{tot} = 10$ C (C being an arbitrary unit of concentration), $k_1 = 7.22$ time^{-1}, $k_2 = 0.63$ time^{-1}, $k_5 = 0.36$ C^{-1} time^{-1}, $k_6 = 0.40$ time^{-1}, $k_7 = 0.63$ C^{-1} time^{-1}, $k_8 = 0.25$ time^{-1}, $k_9 = 0.057$ C^{-1} time^{-1}, $k_{10} = 0.17$ time^{-1}, $k_{12} = 0.5$ time^{-1}, and $k_{16} = 1.71$ time^{-1}. The degradation rate k_{11} is used as the bifurcation parameter.

Translating bistable network models into the experimental data mining format

Because limitations in the data-gathering techniques do not allow for the identification of interactions between heterodimers and DNA (reactions *m*, *p*, *s*, and *v*) with any certainty, we did not consider these reactions when searching for networks. Similarly, it cannot be determined from the data whether the TFs bind to DNA as monomers or dimers; for example, although we distinguish in our modeling framework between P_1 binding to X_1 and P_1P_1 binding to X_1, that resolution does not exist in the experimental data. We therefore generated new 'experimental evidence' labels for pairs of reactions that cannot be distinguished: reactions *b* and *n* are referred to with label <u>*b*</u>, *c* and *o* with label <u>*c*</u>, *a* and *l* with label <u>*a*</u>, and *d* and *q* with label <u>*d*</u>. Lastly, for most of the pairs of TFs and promoters which are known to associate, the effect of that association on target gene expression (activation or repression) is usually unknown.

Information suggestive of a particular effect, gathered from a large number of TF deletion strains and listed in Supplementary Table S4, was viewed as only supplementary in the process of network discovery. With these experimental limitations in mind, networks may be ‘translated’ from their theoretical description into one that takes the limitations into account. For example, the minimal bistable networks may be written as (theoretical name ⇔ translated name) are:

1) *kqw ⇔ dk(w)*
2) *ckn ⇔ bck*
3) *bcdh ⇔ bcd(h)*
4) *abejp ⇔ ab(e)j*
5) *bfjpv ⇔ b(f)j*
6) *jmpsv ⇔ j*
7) *ikno ⇔ bcik*
8) *jknptv ⇔ bjk(t)*
9) *aejknp ⇔ ab(e)jk*
10) *jkmnps ⇔ bjk*
11) *dhjknp ⇔ bd(h)jk*

Labels in parenthesis indicate a reaction that is supplementary to the network discovery.
